# Memoir on Iliac Abscesses and Tumours

**Published:** 1830-01-01

**Authors:** 


### MEMOIR ON ILIAC ABSCESSES AND TUMOURS. By M. TEALLIER, M.D.

[Published by the Med. Soc. of Paris.]

Our readers will remember the Memoir of Messrs. Husson and Dance on this subject, of which we have given an ample account in this Journal. Subsequently M. Menieres brought forward several illustrations of iliac abscesses and tumours, which we also analyzed, together with the clinical observations of M. Dupuytren.* It is remarkable that so many cases of this disease should have been collected in the course of eighteen months or two years, although it is one which was never before described. Without denying that new diseases originate from time to time, we may fairly conclude that most of them arise from the increased cultivation of morbid anatomy and symptomatology. M. Teallier has, in the memoir before us, added four or five cases to the common stock, and as that stock is yet comparatively small, we shall give a brief account of these cases in this article.

*Case* 1. M. T. de Clermont-Ferrand, aged 25 years, of delicate constitution, and subject to intestinal inflammations for two years previously, took a journey of pleasure to Paris in the month of June, and there walked about very much during the first few days. On returning home one evening from the theatre, he was suddenly seized with acute pain in the right iliac fossa, accompanied by vomiting of bile. M. T. saw the patient next morning. He had passed a very bad night—was constipated—his abdomen tense and tender on pressure—pulse quick. Twenty leeches to the iliac region—poultices—fomentations—warm bath. By these means the symptoms were mitigated, and on the 3d day examination discovered an oblong tumour, the size of a hen's egg, behind and rather above the cæcum. The skin over it was shining, and it was very tender to the touch. By repeated applications of leeches, fomentations, baths, and low diet, the pain and inflammatory symptoms subsided, and the tumour entirely disappeared.

*Case* 2. Madam Tenaillon, 24 years of age, of good constitution, was confined in the month of March, and had a good time; but the accouchement was followed by painful colic for many days, and the lacteal secretion was impeded. An emetic was given, and augmented her sufferings. In about three weeks the patient made an attempt to walk out, but the intestinal pains were greatly exasperated, and she took to her bed. M. Teallier was summoned to her assistance. He was struck with the size of the abdomen, as well as its tenderness on pressure. No examination could be borne at this time; but by general and local bleeding the inflammatory symptoms were mitigated, and an examination was rendered practicable at the end of eight days. An

---

* See No. XVIII. p. 274, et seq.

oblong tumour was then discovered in the left iliac fossa, to which place the patient had usually referred the greater part of her sufferings, the size of a turkey's egg, and very painful on pressure. It appeared to be distinct from the intestine, which was felt to glide over it. In proportion as the general abdominal tumefaction subsided, the tumour became more salient. Repeated applications of leeches were made—the bowels kept open—the tumour fomented and poulticed—warm baths used night and morning. By these means the general inflammatory symptoms were entirely reduced; but no impression was made on the tumour, in which a throbbing was now felt by the patient, that caused fear of suppuration in the mind of Dr. Teallier. A seton was inserted over the tumour, and a copious drain of purulent secretion established. The size of the swelling visibly declined, and was totally gone at the end of six months.

*Case* 3. This was a gentleman, 56 years of age, who had led a dissolute and irregular life, and had occasionally suffered from what was considered gravel. In the month of April, 1826, he was seized with very acute pains in the right loin, extending down to the crural arch and testicle, the latter being very much retracted. There was much tenderness on pressure in the above region, and frequent inclination to make water. General and local bleedings were employed several times, with warm baths and diluent drinks. The patient was so exceedingly fat that no satisfactory examination could be made. After fifteen days of severe suffering, he passed all at once by urine a large quantity of inodorous pus, by which he was immediately relieved—and quickly recovered. One year afterwards nearly the same train of symptoms recurred, and after many days of great pain in the right iliac region, an abscess burst while at stool, and a pint and a half of pus was discharged by the rectum. At first he was relieved, but a draining of matter continued—hectic fever came on, and the patient died. Permission to open the body could not be obtained.

*Case* 4. Madame Amans, aged 31 years, became pregnant for the first time in her 30th year. During utero-gestation she suffered pain in the right iliac region, which was exasperated by the movements of the child. She was safely delivered, but the placenta adhered, and was removed by the hand. The belly remained swollen and tender. At the end of a month the lochia were nearly as abundant as immediately after the accouchement, and diarrhœa came on. There was much pain in the right iliac fossa, and she walked lame on that side. A physician who was called in prescribed bark and wine. The symptoms increased and Dr. Teallier was summoned. This was seven weeks after the accouchement. The patient had kept her bed for some days. The abdomen was large and tender—the stools frequent. There was an inflammatory swelling above the right groin, which extended to the thigh and leg, in the direction of the lymphatic vessels, which were hard and tense as cords, very painful on pressure. The other thigh was unaffected at this time, but became slightly so in the sequel. Leeches were applied to the inflamed parts, and fomentations were used, with the most rigid diet. This plan was followed by a considerable amelioration of the symptoms, and the inflammation subsided in the line of the lymphatics in the course of a fortnight; but the pain in the iliac fossa was renewed with increased violence, and spread to all the neighbouring parts. Fever was lighted up, and general bleeding was prescribed, while 30 leeches were applied to the groin. Twenty leeches were twice afterwards put to the same place. The pains became concentrated in the right iliac fossa, and abandoned the surrounding parts. There was now little doubt that the inflammation was terminating in suppuration. A tumour, the size of a fist, was now perceptible in the iliac region, hard and painful to the touch. It went on rapidly to suppuration, and was opened on the 12th of January, just above the ligament of Fallopius. There was a large discharge of pus, blood, and flocculent matters.

More than a pint flowed away, and about the same quantity was left, when the wound was closed for a time. On being opened again a large quantity issued forth, and this repeatedly took place afterwards. The discharge gradually diminished, and health appeared to be on the verge of restoration, when a sudden attack of anasarca came on, rising rapidly from the lower extremities over the whole body. The abscess now burst open again, and a large quantity of serum came forth. From this time all the bad symptoms subsided, and the patient was pronounced cured by the end of February.—*Journ. Gen. de Medecine.*

---

VIII.

